# Exact matching of trabectome-mediated ab interno trabeculectomy to conventional trabeculectomy with mitomycin C followed for 2 years

**DOI:** 10.1007/s00417-020-05031-w

**Published:** 2020-12-02

**Authors:** A. Strzalkowska, P. Strzalkowski, Y. Al Yousef, F. Grehn, J. Hillenkamp, Nils A. Loewen

**Affiliations:** Department of Ophthalmology, University Hospital Würzburg, Josef-Schneider-Straße 11, 97080 Würzburg, Germany

**Keywords:** Trabeculectomy, Ab interno trabeculectomy, Trabectome, Exact matching

## Abstract

**Purpose:**

We used exact matching for a highly balanced comparison of ab interno trabeculectomy (AIT) with the trabectome to trabeculectomy with mitomycin C (TRAB).

**Methods:**

A total of 5485 patients who underwent AIT were exact-matched to 196 TRAB patients by baseline intraocular pressure (IOP), number of glaucoma medications, and glaucoma type. Nearest-neighbor–matching was applied to age. Success was defined as a final IOP of less than 21 mmHg, IOP reduction of at least 20% reduction from baseline, and no secondary surgical interventions. Outcomes were measured at 1, 3, 6, 12, 18, and 24 months.

**Results:**

A total of 165 AIT could be matched to 165 TRAB. The mean baseline IOP was 22.3 ± 5.6 mmHg, and the baseline number of glaucoma medications was 2.7 ± 1.1 in both groups. At 24 months, IOP was reduced to 15.8 ± 5.2 mmHg in AIT and 12.4 ± 4.7 mmHg in TRAB. IOP was lower than baseline at all visits (*p* < 0.01) and lower in TRAB than AIT (*p* < 0.01). Glaucoma medications were reduced to 2.1 ± 1.3 in AIT and 0.2 ± 0.8 in TRAB. Compared to baseline, patients used fewer drops postoperatively (*p* < 0.01) and more infrequently in TRAB than in AIT (*p* > 0.01). Secondary surgical interventions had the highest impact on success and became necessary in 15 AIT and 59 TRAB patients. Thirty-two challenging events occurred in TRAB and none in AIT.

**Conclusion:**

Both AIT and TRAB reduced IOP and medications. This reduction was more significant in TRAB but at the expense of four times as many secondary interventions.

**Supplementary Information:**

The online version contains supplementary material available at 10.1007/s00417-020-05031-w.

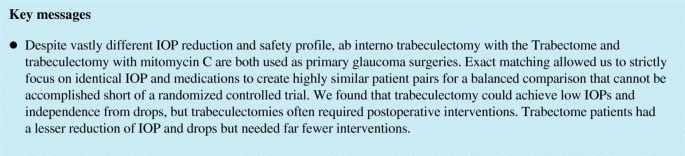

## Introduction

Trabeculectomy (TRAB), first performed in patients in 1961 by Sugar [1] and made more effective by the addition of mitomycin C in 1990 [2], continues to play a central role in surgical glaucoma treatment. However, postoperative challenges and complications can occur in up to 77–78% of patients [3–6]. This concern led to the development of nonpenetrating and microincisional glaucoma surgeries (MIGS) [7]. One of the MIGS, ab interno trabeculectomy (AIT), is now often used both as a primary [8] and a secondary surgery [9] due to its proven, relative effectiveness in a range of glaucoma severity, including severe glaucoma [10–12]. AIT can also be performed after a failed trabeculectomy [13] or tube shunt [14]. This is surprising because TRAB allows aqueous humor to bypass the conventional outflow system, causing Schlemm’s canal and collector channels to atrophy [15, 16].

In theory, the IOP that AIT can achieve is limited by the episcleral venous pressure of 8 mmHg [17], yet most studies report IOPs around 15 mmHg. In contrast, TRAB can achieve very low IOPs, even below episcleral venous pressure, and eliminate drops [18, 19]. Hypotony [20, 21] is a feared consequence of too much pressure reduction. Phaco-AIT has been compared to Phaco-TRAB in a randomized controlled trial but was stopped, with only 19 patients enrolled due to lack of clinical equipoise: TRAB was more complication prone than AIT, yet IOPs were similar [22]. There is scant data that directly compare TRAB to AIT. TRAB is a filtering surgery that drains aqueous humor into a subconjunctival reservoir, the bleb, while AIT enhances conventional outflow along its physiological route by removing outflow resistance at the level of the trabecular meshwork. There is currently insufficient data from a direct comparison of TRAB and AIT to decide whether both could be used as a primary glaucoma surgery in similar patients.

To address this unmet need, we applied an advanced method in statistics, exact matching [23–25], and created nearly identical pairs of AIT and TRAB based on IOP and number of topical glaucoma medications while using nearest neighbor matching for age. This design allowed us to compare the fate of highly similar eyes after being treated by these two distinctly different methods. We left out variables on disease severity to strictly focus on IOP and number of medications. The visual field severity and number of preoperative medications do not impact IOP reduction by AIT when they are examined independently of IOP [10–12].

## Patients and methods

### Study design

The study protocol was approved by the local ethics committee of the University of Würzburg, Germany, and performed in accordance with the ethical standards set forth in the 1964 Declaration of Helsinki and the Health Insurance Portability and Accountability Act. Because of its retrospective nature, informed consent was waived. All patients included in the study underwent either ab interno trabeculectomy using the trabectome (AIT) or trabeculectomy with 0.02% mitomycin C (TRAB). An indication for surgery was an above-target IOP despite maximally tolerated topical treatment, as determined by a glaucoma specialist, or a need to reduce glaucoma medications despite stable IOP due to eye drop intolerance. We used data from the Trabectome Study Group database [13, 26] from 2013 to 2018 to increase the chances of an exact match to the TRAB group. TRABs were from between 2007 and 2014 and performed by a single surgeon (FG). A total of 5485 AIT cases were matched to 196 TRAB. Patients younger than 20 years of age with neovascular or uveitic glaucoma were excluded. We recorded medical history, best-corrected visual acuity (BCVA [logMAR]), IOP (Goldmann tonometry [mmHg]), topical glaucoma medications, events leading to failure and serious, vision-threatening complications known from prior trabeculectomy and tube shunt studies, including aqueous misdirection, infection, wound leaks, hypotony maculopathy, choroidal effusions, choroidal hemorrhage, and corneal decompensation. Success was defined as a final IOP ≤ 21 mmHg, IOP reduction of at least 20% from baseline, and no secondary surgical interventions. Needling in the operating room in TRAB was counted as a surgical intervention. The decision to continue glaucoma drops was at the discretion of the treating specialist, as was the decision to advance to a different glaucoma surgery.

### Statistics

Data were described as frequency, percentage, mean ± SD, median, and range. Continuous and categorical variables were compared with the Mann–Whitney *U* test and chi-squared test. Using exact matching, both groups were matched using preoperative IOP, glaucoma medications, type of glaucoma, and using nearest neighbor matching for age [27]. Each unit in AIT was matched using exact matching to all possible control units in TRAB, whereas nearest neighbor matching selected the best match based on the distance to the value in AIT. *P* values of less than 0.05 were considered statistically significant. Mean ± SD was used to express continuous variables. Statistical analyses were performed using *R* [28]. A Kaplan-Meier plot was generated.

### Surgical technique

AIT was performed as described before [29]. Briefly, a 1.6-mm uniplanar, temporal clear corneal incision was created. Under direct gonioscopic visualization, the tip of the trabectome handpiece (MST MicroSurgical Technology, Redmond, WA, USA) was inserted into Schlemm’s canal. The TM was ablated counterclockwise, followed by clockwise ablation with a total length of about 120° [30–32]. Ablation was started with the power set to 0.8 mW and increased as necessary. The handpiece was withdrawn from the anterior chamber.

In TRAB, the eye was rotated downward with a traction suture, as described before [33]. A 5-mm limbus–based peritomy was created at the anatomic 12 o’clock position. A subtenon pocket was fashioned to accommodate a sponge soaked with mitomycin C (MMC) at a concentration of 0.2 mg/ml for 3 min. A 3.5-mm × 4-mm scleral flap was created. A 0.8 mm × 2 mm sclerotrabecular block was excised to enter the anterior chamber. A peripheral iridectomy was made. The scleral flap was secured with 10–0 nylon to allow visible percolation of aqueous, and the conjunctiva was closed with an interlocking running suture resulting in a diffusely forming bleb. All patients received daily subconjunctival injections of 2 mg of 5 fluorouracil (5-FU) for 1 week except when IOP was at or below 5 mmHg or the Seidel test was positive for a bleb leak.

In both AIT and TRAB, postoperative treatment consisted of a combination of a topical antibiotic for 1 week and a steroid tapered over 4 weeks. Glaucoma medications were stopped on the day of surgery and restarted as needed.

## Results

Using exact matching, 165 AIT were paired with 165 TRAB. The baseline IOP and glaucoma medications in AIT and TRAB were identical. IOP was 22.3 ± 5.6 mmHg, and medications were 2.7 ± 1.1. The demographic characteristics are presented in Table 1. AIT patients had an average age of 50 ± 15 years, significantly younger than TRAB 67 ± 11 (*p* < 0.01). Despite their younger age, 60% of AIT patients had prior surgeries, many more than TRAB, with only one prior trabeculectomy (0.6%). Prior procedures in AIT included 13 (8%) ALT, 38 (23%) SLT, 20 (12%) cataract surgeries, 9 (5%) other surgeries, as well as major glaucoma surgeries consisting of 16 (10%) trabeculectomies and 4 (2%) tube shunts. Forty-six (28%) AIT operations were combined with phacoemulsification. The prevalence of diabetes mellitus in our TRAB population was 23.8% and in our AIT population 21.9%.

**Table 1:** Demographics (* = *p* < 0.05)

	AIT (*n* = 165)	TRAB (*n* = 165)
Age*		
Mean ± SD	50 ± 15	67 ± 11
Range	(16, 87)	(25, 93)
Gender		
Female	72 (44%)	86 (52%)
Male	92 (56%)	79 (48%)
Diagnosis		
Primary open angle glaucoma	130 (79%)	130 (79%)
Pseudoexfoliation glaucoma	32 (19%)	32 (19%)
Pigment dispersion glaucoma	3 (2%)	3 (2%)
Prior procedures and surgeries*		
Argon laser trabeculoplasty (ALT)	13 (8%)	48 (29%)
Selective laser trabeculoplasty (SLT)	38 (23%)	12 (7%)
Cataract	20 (12%)	–
Trabeculectomy	16 (10%)	1 (0.6%)
Shunt	4 (2%)	–
Other	9 (5%)	–
Combined surgeries*		
Combined with phaco	46 (28%)	–
Combined with other	4 (2%)	–

**Fig. 1:**
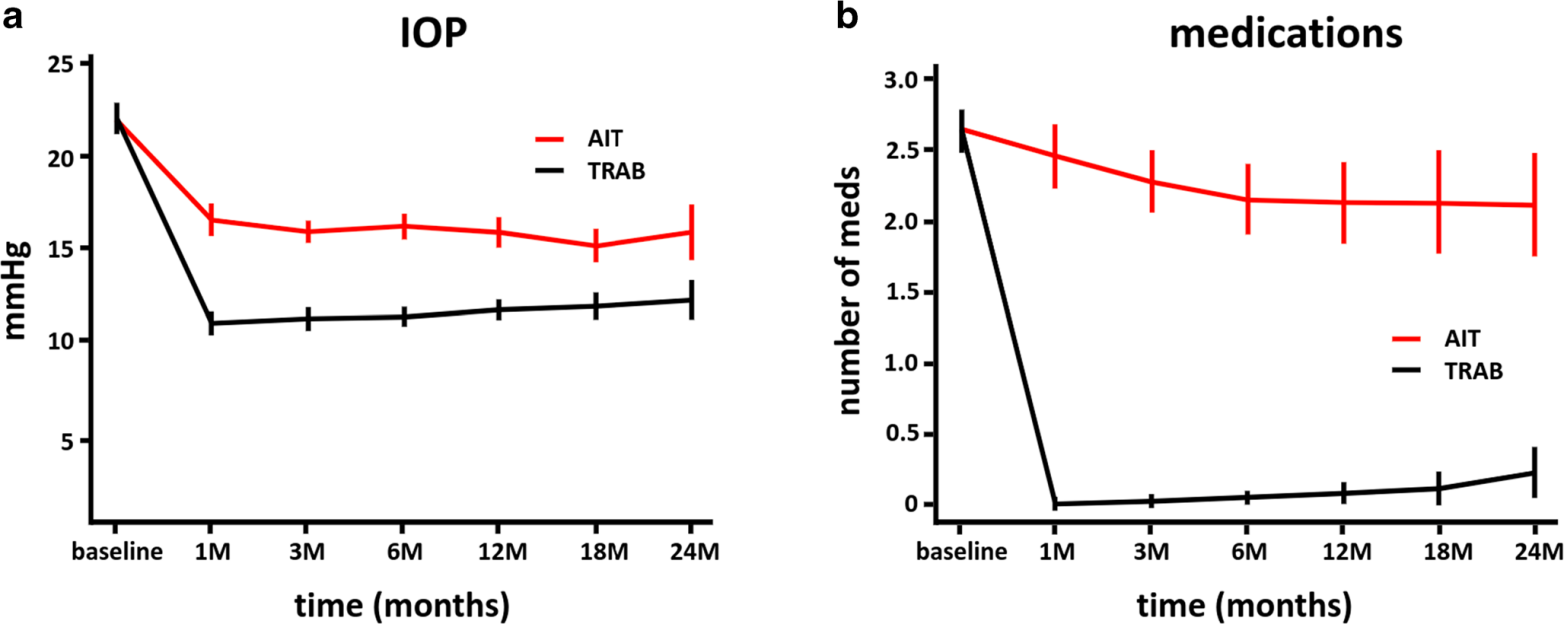
IOP and number of medications for AIT and TRAB. **a** IOP decreased to a relatively stable level in AIT and slightly trended up in TRAB. **b** Glaucoma medications continued to decrease for 6 months and remained at this level and slightly trended up in TRAB after a sharp drop at 1 month (mean ± SEM; subject count at each time point)

At 1 month, IOP decreased to 16.7 ± 5.6 in AIT and 11.1 ± 4.0 in TRAB (Table 2; Fig. 1a, all-time points *p* < 0.01). IOP stayed relatively similar throughout the study, trending towards 15.8 ± 5.2 mmHg in AIT and 12.4 ± 4.7 in TRAB 24 months. Medications had declined from 2.7 ± 1.1 in each group to 2.4 ± 1.5 in AIT and 0.0 ± 0.2 in TRAB at 1 month. At 24 months, they were at 2.2 ± 1.3 in AIT and 0.2 ± 0.8 in TRAB (all time points *p* < 0.01). AIT had a significantly higher IOP and number of medications throughout the study (*p* < 0.01). In TRAB, the medication reduction was significant (*p* < 0.01, Table 2; Fig. 1b) at all time points of the study compared to baseline. In AIT, the medication reduction was small and only became statistically significant after 3 months (after 3 months *p* < 0.05).

**Table 2:** Mean IOP and number of medication for AIT and TRAB at each follow-up time point (Welch two-sample *t* test, significance level set at ≤ 0.05)

	IOP (mean ± SD)			Rx (mean ± SD)		
Time	AIT	TRAB	*P* value	AIT	TRAB	*P* value
baseline	22.3 ± 5.6	22.3 ± 5.6		2.7 ± 1.1	2.7 ± 1.1	
1 month	16.7 ± 5.6	11.1 ± 4.0	< 0.01*	2.4 ± 1.5	0.0 ± 0.2	< 0.01*
3 months	16.0 ± 3.9	11.4 ± 3.9	< 0.01*	2.3 ± 1.5	0.0 ± 0.3	< 0.01*
6 months	16.3 ± 4.1	11.5 ± 3.4	< 0.01*	2.1 ± 1.4	0.0 ± 0.4	< 0.01*
12 months	16.0 ± 3.9	11.9 ± 3.3	< 0.01*	2.1 ± 1.3	0.1 ± 0.4	< 0.01*
18 months	15.3 ± 3.4	12.1 ± 3.4	< 0.01*	2.2 ± 1.4	0.1 ± 0.5	< 0.01*
24 months	15.8 ± 5.2	12.4 ± 4.7	< 0.01*	2.2 ± 1.3	0.2 ± 0.8	< 0.01*

When all interventions were counted towards surgical failure, success was achieved in 93% of AIT and 58% of TRAB eyes (difference *p* < 0.01, Supplementary Fig. 1). Additional surgery was needed in 15 (9%) of AIT and 59 (36%) of TRAB. In TRAB, 24 (14.9%) patients needed bleb needling, often considered a minor secondary surgical intervention, 33 (20.0%) an open bleb revision, one iris repositioning (0.6%) and one cyclodestruction (0.6%). In AIT, 15 of 18 patients with high IOP received a tube shunt. Success criteria were not fulfilled in 18 AIT (11%) and one TRAB patient (0.6%, Table 3**)**. In TRAB, 17 (10%) eyes had a shallow anterior chamber, 16 (10%) developed a cataract, 7 (4%) choroidal effusion, 6 (4%) with bleb leaks, and 3 (2%) with iris adhesion.

**Table 3:** Number of intervention for AIT and TRAB within 24 months

Group	AIT	TRAB
Patients with failure	33 (20%)	60 (36.6%)
due to intervention including bleb needling		59 (36%)
due to intervention excluding bleb needling	15 (9%)	35 (14.9%)
due to high IOP	18 (11%)	1 (0.6%)

An additional intervention was needed in 15 (9%; 45% of all failures) of AIT and 59 (36%; 98% of all interventions) of TRAB (Table 3). In AIT, 15 (9%) patients (45% of all interventions) received a tube shunt. In TRAB, one patient needed a secondary glaucoma surgery. However, 24 (14.9%) TRAB patients (41% of all interventions) needed bleb needling in the operating room, 33 eyes (20.0%, 56% all interventions) needed an open scleral flap revision most of which resulted in successful IOP control, one cyclodestruction (0.6%, 1.5% of all failures) and one iris repositioning (0.6%, 1.5% of all failures). Seventeen (10%) TRAB eyes had a shallow anterior chamber, 16 (10%) developed a cataract, 7 (4%) choroidal effusion, 6 (4%) bleb leaks, and 3 (2%) iris adhesion.

Failure to control IOP (final IOP ≤ 21 mmHg, IOP reduction of at least 20% from baseline) was more common in AIT, where it occurred in 18 (10.9%) compared to only one (0.6%) in TRAB.

## Discussion

This study aimed to compare IOP and glaucoma medication reduction by two distinctly different glaucoma surgeries, AIT and TRAB, commonly used as primary surgical treatments. We wanted this comparison to be agnostic to disease severity and instead generate a highly balanced comparison, strictly focusing on IOP and medications in our exact matching algorithm. The preoperative IOP, and its modification through glaucoma medications, is the single most important predictor of IOP reduction by AIT [34]. In contrast, the visual field status or preoperative medications on their own do not have a significant impact on postoperative IOP reduction [10–12]. Matching is a nonparametric method designed to control confounding [35]. Propensity score matching would be well suited to compare such dissimilar datasets [36, 37], while exact matching is more commonly used to compare similar data [23, 38]. However, exact matching allowed us in this study to more carefully examine two of the most important variables in glaucoma care, IOP, and eye drops needed to achieve this IOP.

Techniques and mechanisms of IOP reduction of AIT and TRAB are vastly different. TRAB lowers IOP by allowing aqueous humor to bypass the conventional outflow system and drain into a bleb, a subtenon reservoir, from which aqueous is removed by lymphatics, absorption, and transconjunctival diffusion [39]. It is well established [40, 41] that trabeculectomies can lower IOP and reduce medications profoundly, but have a higher risk of complications and often need additional interventions. In contrast to that, AIT removes a primary substrate of outflow resistance, the trabecular meshwork [29]. Hypotony-related complications do not normally occur in AIT because the IOP cannot be lower than the episcleral venous pressure. Recent studies have demonstrated that the average IOP after AIT is higher than one would expect based on the 8 mmHg present in the episcleral veins [34]. There is new evidence of a post-trabecular outflow resistance [42–44] that could cause AIT to fail but would not affect the results of TRAB.

Trabeculectomy is known to increase the cataract progression rate to up to twice that of controls [45, 46]. In our study, TRAB had a higher rate of cataract progression than AIT. AIT patients were younger, 20 already had cataract surgery, and 46 had cataract removal combined with AIT. It is possible that trabeculectomy surgeons focused on IOP and avoided the additional challenges and risks of cataract surgery in the same session. Combining cataract surgery with AIT does not contribute to IOP reduction, however [47]. In contrast, cataract surgery can lower IOP by 1.5–3 mmHg when done on its own [48–50] or when combined with a trabecular bypass stent [51], due to a trabeculoplasty-like effect [23, 52]. However, too much trabecular meshwork is removed in AIT to be affected by this, and more circumferential flow is generated compared to trabecular bypass stents [53].

Prior laser procedures and incisional glaucoma surgeries can jeopardize the success of AIT. AIT patients had a considerably higher rate of prior surgeries, putting them at a disadvantage in our study. Argon laser trabeculoplasty (ALT) causes coagulative damage to the trabecular meshwork and Schlemm’s canal [54]. These create perceptible stops during AIT that likely limit circumferential flow. Selective laser trabeculoplasty (SLT) does not do that [55].

Consequently, when SLT fails to lower IOP, it could indicate that the distal outflow system is dysfunctional, reducing the chances that AIT will work. This theoretical consideration was not seen in Klamann et al.’s [56] study, however. A failed prior tube shunt or trabeculectomy may also reduce chances of a successful AIT as there is histological evidence that Schlemm’s canal and collector channels atrophy [15, 16]. This concern did equally not materialize in two studies that found that there was a reasonable IOP reduction by AIT after failed tubes [14] and trabeculectomies [13].

TRAB patients had a significantly better reduction of IOP and medications at 2 years in our study, whereas patients in AIT still required more than two glaucoma drops on average. While 15 (9%) of patients in AIT needed another major glaucoma operation (tube shunt), there was only one further glaucoma procedure (cyclophotocoagulation) in TRAB. This advantage of TRAB comes at the price of more intense postoperative care (i.e., postoperative 5-fluorouracil injections) and additional interventions such as bleb needling and sometimes surgical revisions. However, many of these interventions, albeit cumbersome for the patient and the surgeon, often have no negative impact on the final outcome. In addition, postoperative challenges such as choroidal effusion and a shallow anterior chamber often resolve on their own.

Diabetes mellitus can reduce the success rate of trabeculectomy [57]. The prevalence of diabetes mellitus in both our TRAB and AIT population was similar to that in the Advanced Glaucoma Intervention Study with 18.3% [41] which makes our results more comparable. Although our AIT patients were younger than TRAB patients, 28% had concurrent cataract surgery, and 12% were already pseudophakic. None of these conditions would generate a better IOP reduction, as we have shown before [34, 47]. The AIT data used in our study to create exact matches do not represent the experience of a single surgeon or single center but represents the average of multiple centers and surgeons. Surgeons had to submit their first cases as part of a post-market surveillance requirement by the US Food and Drug Administration (FDA) [13, 26]. Likely, most AIT surgeons were still on the learning curve, ranging from 5 to 30 eyes, as observed in an AIT training model [58].

Limitations of our study include its retrospective nature. This shortcoming is countered by exact matching, a robust automated statistical method that creates a highly balanced comparison and reduces confounding [59, 60]. Although the algorithm discarded AIT data, we were fortunate to be able to afford this and create identical IOP and glaucoma eye drop matches with the much more limited number of TRAB so that only 31 were lost. Had we increased the number of matching variables to improve the precision of matching, many more patients would have been excluded, reducing the study sample size and variability of the patient population [60, 61]. Consequently, we applied nearest neighbor matching to age, and AIT patients ended up with a younger average age of 50 ± 15 years compared to TRAB with 67 ± 11 years. Although this age difference is considerable, it would have put AIT only at a slight disadvantage of approximately 0.5 mmHg as predicted by our IOP calculator [34]. Similarly, 95% of our TRAB patients were above the age of 46 years. Briggs et al. [62] showed that an age over 46 years does not affect the IOP lowering effect of TRAB.

Another limitation is that we ignored the individual target IOP to focus on IOP and medications. Using a common definition of success, which considered any secondary surgical intervention [63, 64], AIT had a higher success rate than TRAB. However, many trabeculectomists would not consider needling or revisions a complication as long as the target pressure is achieved eventually. Our TRAB achieved an IOP reduction equal to that in the TVT study but with fewer medications, while our AIT appeared to be similar to the tubes in that study [65].

Our study confirmed that TRAB remains a good choice for patients with low IOP targets and need to reduce drops. This procedure requires patients to accept a high chance of postoperative challenges. Analysis of AIT patients matched to the relatively low preoperative IOP of TRAB patients confirm that AIT is a low-risk procedure that achieves physiological IOPs.

Fortunately, TRAB and AIT do not exclude each other as AIT can be performed after TRAB has failed [13] and vice versa [66].

## Supplementary information

ESM 1 (DOCX 21 kb)

## References

[1] Saul Sugar H (1961). Experimental trabeculectomy in glaucoma *. Am J Ophthalmol.

[2] Chen CW, Huang HT, Bair JS, Lee CC (1990). Trabeculectomy with simultaneous topical application of mitomycin-C in refractory glaucoma. J Ocul Pharmacol.

[3] Hylton C, Congdon N, Friedman D (2003). Cataract after glaucoma filtration surgery. Am J Ophthalmol.

[4] Picht G, Mutsch Y, Grehn F (2001). Nachbetreuung von Trabekulektomien Komplikationen und therapeutische Konsequenzen. Ophthalmologe.

[5] Gedde SJ, Herndon LW, Brandt JD (2007). Surgical complications in the tube versus trabeculectomy study during the first year of follow-up. Am J Ophthalmol.

[6] Bindlish R, Condon GP, Schlosser JD, D’Antonio J (2002). Efficacy and safety of mitomycin-C in primary trabeculectomy: five-year follow-up. Ophthalmology.

[7] Kaplowitz K, Bussel I, Loewen NA, Yanoff M, Duker JS (2013). Minimally Invasive and nonpenetrating glaucoma surgeries. Ophthalmology: expert consult: Online and Print. Elsevier - Health Sciences Division.

[8] Kaplowitz K, Bussel II, Honkanen R (2016). Review and meta-analysis of ab-interno trabeculectomy outcomes. Br J Ophthalmol.

[9] Jordan JF, Wecker T, van Oterendorp C (2013). Trabectome surgery for primary and secondary open angle glaucomas. Graefes Arch Clin Exp Ophthalmol.

[10] Loewen RT, Roy P, Parikh HA (2016). Impact of a glaucoma severity index on results of trabectome surgery: larger pressure reduction in more severe glaucoma. PLoS One.

[11] Roy P, Loewen RT, Dang Y (2017). Stratification of phaco-trabectome surgery results using a glaucoma severity index in a retrospective analysis. BMC Ophthalmol.

[12] Dang Y, Roy P, Bussel II (2016). Combined analysis of trabectome and phaco-trabectome outcomes by glaucoma severity. F1000Res.

[13] Bussel II, Kaplowitz K, Schuman JS (2015). Outcomes of ab interno trabeculectomy with the trabectome after failed trabeculectomy. Br J Ophthalmol.

[14] Mosaed S, Chak G, Haider A (2015). Results of trabectome surgery following failed glaucoma tube shunt implantation: cohort study. Medicine.

[15] Lütjen-Drecoll E (1999). Functional morphology of the trabecular meshwork in primate eyes. Prog Retin Eye Res.

[16] Johnson DH, Matsumoto Y (2000). Schlemm’s canal becomes smaller after successful filtration surgery. Arch Ophthalmol.

[17] Dang Y, Wang C, Shah P et al (2018) Outflow enhancement by three different ab interno trabeculectomy procedures in a porcine anterior segment model. Graefes Arch Clin Exp Ophthalmol. 10.1007/s00417-018-3990-0 10.1007/s00417-018-3990-0 PMC7804591 29721662

[18] Iverson SM, Schultz SK, Shi W (2016). Effectiveness of single-digit IOP targets on decreasing global and localized visual field progression after filtration surgery in eyes with progressive normal-tension glaucoma. J Glaucoma.

[19] Schultz SK, Iverson SM, Shi W, Greenfield DS (2016). Safety and efficacy of achieving single-digit intraocular pressure targets with filtration surgery in eyes with progressive normal-tension glaucoma. J Glaucoma.

[20] Maruyama K, Shirato S (2008). Efficacy and safety of transconjunctival scleral flap resuturing for hypotony after glaucoma filtering surgery. Graefes Arch Clin Exp Ophthalmol.

[21] Saeedi OJ, Jefferys JL, Solus JF (2014). Risk factors for adverse consequences of low intraocular pressure after trabeculectomy. J Glaucoma.

[22] Ting JLM, Rudnisky CJ, Damji KF (2018) Prospective randomized controlled trial of phaco-trabectome versus phaco-trabeculectomy in patients with open angle glaucoma. Can J Ophthalmol. 10.1016/j.jcjo.2018.01.033 10.1016/j.jcjo.2018.01.033 30502982

[23] Esfandiari H, Taubenslag K, Shah P (2019). Two-year data comparison of ab interno trabeculectomy and trabecular bypass stenting using exact matching. J Cataract Refract Surg.

[24] Haubold B, Börsch-Haubold A, Haubold B, Börsch-Haubold A (2017). Exact matching. Bioinformatics for evolutionary biologists: a problems approach.

[25] Akil H, Chopra V, Huang A (2016). Clinical results of ab interno trabeculotomy using the trabectome in patients with pigmentary glaucoma compared to primary open angle glaucoma. Clin Exp Ophthalmol.

[26] Bussel II, Kaplowitz K, Schuman JS (2015). Outcomes of ab interno trabeculectomy with the trabectome by degree of angle opening. Br J Ophthalmol.

[27] King G Nearest Neighbor Matching. https://r.iq.harvard.edu/docs/matchit/2.4-15/Nearest_Neighbor_Match.html. Accessed 8 Jun 2018

[28] Core Team R (2018). R: a language and environment for statistical computing.

[29] Fallano K, Bussel I, Kagemann L (2017). Training strategies and outcomes of *ab interno* trabeculectomy with the trabectome. F1000Res.

[30] Francis BA, Minckler D, Dustin L (2008). Combined cataract extraction and trabeculotomy by the internal approach for coexisting cataract and open-angle glaucoma: initial results. J Cataract Refract Surg.

[31] Kaplowitz K, Loewen NA, Samples JR, Ahmed IIK (2014). Minimally invasive glaucoma surgery: trabeculectomy ab interno. Surgical Innovations in Glaucoma.

[32] Kaplowitz K, Bussel I, Loewen NA (2015) Trabeculectomy by internal approach: trabectome. In: Francis B, Sarkisian S, Tan J (eds) Minimally Invasive Glaucoma Surgery: the Science and the Practice. Thieme, pp 82–91

[33] Marquardt D, Lieb WE, Grehn F (2004). Intensified postoperative care versus conventional follow-up: a retrospective long-term analysis of 177 trabeculectomies. Graefes Arch Clin Exp Ophthalmol.

[34] Neiweem AE, Bussel II, Schuman JS (2016). Glaucoma surgery calculator: limited additive effect of phacoemulsification on intraocular pressure in ab interno trabeculectomy. PLoS One.

[35] Blackwell M, Iacus S, King G, Porro G (2009). Cem: coarsened exact matching in Stata. Stata J.

[36] Esfandiari H, Shazly TA, Waxman SA (2018). Similar performance of trabectome and ahmed glaucoma devices in a propensity score-matched comparison. J Glaucoma.

[37] Kostanyan T, Shazly T, Kaplowitz KB (2017). Longer-term Baerveldt to trabectome glaucoma surgery comparison using propensity score matching. Graefes Arch Clin Exp Ophthalmol.

[38] Al Yousef Y, Strzalkowska A, Hillenkamp J et al (2020) Comparison of a second-generation trabecular bypass (iStent inject) to ab interno trabeculectomy (Trabectome) by exact matching. Graefes Arch Clin Exp Ophthalmol. 10.1007/s00417-020-04933-z 10.1007/s00417-020-04933-z PMC7677264 32960322

[39] Yin X, Cai Q, Song R (2018). Relationship between filtering bleb vascularization and surgical outcomes after trabeculectomy: an optical coherence tomography angiography study. Graefes Arch Clin Exp Ophthalmol.

[40] Gedde SJ, Feuer WJ, Lim KS et al (2019) Treatment outcomes in the primary tube versus trabeculectomy (PTVT) study after three years of follow-up. Ophthalmology. 10.1016/j.ophtha.2019.10.002 10.1016/j.ophtha.2019.10.002 31727428

[41] Investigators AGIS (2002). The advanced Glaucoma intervention study (AGIS): 11. Risk factors for failure of trabeculectomy and argon laser trabeculoplasty. Am J Ophthalmol.

[42] Chen S, Waxman S, Wang C (2020). Dose-dependent effects of netarsudil, a rho-kinase inhibitor, on the distal outflow tract. Graefes Arch Clin Exp Ophthalmol.

[43] Waxman S, Wang C, Dang Y (2018). Structure-function changes of the porcine distal outflow tract in response to nitric oxide. Invest Ophthalmol Vis Sci.

[44] McDonnell F, Dismuke WM, Overby DR, Stamer WD (2018). Pharmacological regulation of outflow resistance distal to Schlemm’s canal. Am J Phys Cell Phys.

[45] Musch DC, Gillespie BW, Niziol LM (2006). Cataract extraction in the collaborative initial glaucoma treatment study: incidence, risk factors, and the effect of cataract progression and extraction on clinical and quality-of-life outcomes. Arch Ophthalmol.

[46] AGIS (Advanced Glaucoma Intervention Study) Investigators (2001). The advanced glaucoma intervention study: 8. Risk of cataract formation after trabeculectomy. Arch Ophthalmol.

[47] Parikh HA, Bussel II, Schuman JS (2016). Coarsened exact matching of phaco-trabectome to trabectome in phakic patients: lack of additional pressure reduction from phacoemulsification. PLoS One.

[48] Mansberger SL, Gordon MO, Jampel H (2012). Reduction in intraocular pressure after cataract extraction: the ocular hypertension treatment study. Ophthalmology.

[49] Yang HS, Lee J, Choi S (2013). Ocular biometric parameters associated with intraocular pressure reduction after cataract surgery in normal eyes. Am J Ophthalmol.

[50] Shingleton BJ, Pasternack JJ, Hung JW, O’Donoghue MW (2006). Three and five year changes in intraocular pressures after clear corneal phacoemulsification in open angle glaucoma patients, glaucoma suspects, and normal patients. J Glaucoma.

[51] Samuelson TW, Katz LJ, Wells JM (2011). Randomized evaluation of the trabecular micro-bypass stent with phacoemulsification in patients with glaucoma and cataract. Ophthalmology.

[52] Wang N, Chintala SK, Fini ME, Schuman JS (2003). Ultrasound activates the TM ELAM-1/IL-1/NF-kappaB response: a potential mechanism for intraocular pressure reduction after phacoemulsification. Invest Ophthalmol Vis Sci.

[53] Parikh HA, Loewen RT, Roy P (2016). Differential canalograms detect outflow changes from trabecular micro-bypass stents and ab interno trabeculectomy. Sci Rep.

[54] Johnson DH (2007). Histologic findings after argon laser trabeculoplasty in glaucomatous eyes. Exp Eye Res.

[55] Latina M, Deleon J (2005). Selective laser trabeculoplasty. Ophthalmol Clin N Am.

[56] Klamann MKJ, Gonnermann J, Maier A-KB (2014). Influence of selective laser trabeculoplasty (SLT) on combined clear cornea phacoemulsification and trabectome outcomes. Graefes Arch Clin Exp Ophthalmol.

[57] Law SK, Hosseini H, Saidi E (2013). Long-term outcomes of primary trabeculectomy in diabetic patients with primary open angle glaucoma. Br J Ophthalmol.

[58] Dang Y, Waxman S, Wang C (2017). Rapid learning curve assessment in an ex vivo training system for microincisional glaucoma surgery. Sci Rep.

[59] Burden A, Roche N, Miglio C (2017). An evaluation of exact matching and propensity score methods as applied in a comparative effectiveness study of inhaled corticosteroids in asthma. Pragmat Obs Res.

[60] Fullerton B, Pöhlmann B, Krohn R (2016). The comparison of matching methods using different measures of balance: benefits and risks exemplified within a study to evaluate the effects of German disease management programs on long-term outcomes of patients with type 2 diabetes. Health Serv Res.

[61] Stuart EA (2010). Matching methods for causal inference: a review and a look forward. Stat Sci.

[62] Briggs MC, Jay JL (1999). Age over 46 years does not affect the pressure lowering effect of trabeculectomy in primary open angle glaucoma. Br J Ophthalmol.

[63] Gedde SJ, Schiffman JC, Feuer WJ (2005). The tube versus trabeculectomy study: design and baseline characteristics of study patients. Am J Ophthalmol.

[64] Shaarawy TM, Sherwood MB, Grehn F (2009). WGA guidelines on design and reporting of glaucoma surgical trials.

[65] Gedde SJ, Schiffman JC, Feuer WJ (2012). Treatment outcomes in the tube versus trabeculectomy (TVT) study after five years of follow-up. Am J Ophthalmol.

[66] Jea SY, Mosaed S, Vold SD, Rhee DJ (2012). Effect of a failed trabectome on subsequent trabeculectomy. J Glaucoma.

