# Supplementary material for: Exact matching of trabectome-mediated ab interno trabeculectomy to conventional trabeculectomy with mitomycin C followed for 2 years
Source: Graefes Arch Clin Exp Ophthalmol. 2020 Dec 2;259(4):963–70. doi: 10.1007/s00417-020-05031-w (PMC8016747; doi:10.1007/s00417-020-05031-w)
Supplement: Supplementary file 1 — (DOCX 21 kb) [file 417_2020_5031_MOESM1_ESM.docx]

## Supplemental figure 1


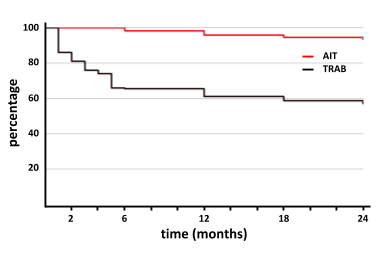


**Supplemental figure 1.** Kaplan-Meier curve indicating how many eyes fulfilled the success criteria (IOP ≤ 21 mm Hg, IOP reduction of at least 20% reduction from baseline, and no secondary surgical interventions including needling in the OR).
